# Novel Minimal Absent Words Detected in Influenza A Virus

**DOI:** 10.3390/v17050659

**Published:** 2025-04-30

**Authors:** Elif Zülal Bigiş, Elif Yıldız, Anna Tagka, Athanasia Pavlopoulou, George P. Chrousos, Styliani Geronikolou

**Affiliations:** 1 Izmir Biomedicine and Genome Center, 35340 Balçova, Izmir, Türkiye; 2 Izmir International Biomedicine and Genome Institute, Dokuz Eylül University, 35340 Balçova, Izmir, Türkiye; 3 First Department of Dermatology-Venereology, Medical School, National and Kapodistrian University of Athens, Andreas Syggros Hospital of Venereal & Dermatological Diseases, 10527 Athens, Greece; 4 University Research Institute of Maternal & Child Health & Precision Medicine, Medical School, National and Kapodistrian University of Athens, Levadeias 8, 10527 Athens, Greece

**Keywords:** influenza A virus surveillance, avian influenza, H1N1, H5N1, phylogeny, hemagglutinin, minimal absent words

## Abstract

Influenza is a communicable disease caused by RNA viruses. Strains A (affecting animals, humans), B (affecting humans), C (affecting rarely humans and pigs), and D (affecting cattle) comprise a variety of substrains each. Influenza A strain, affecting both humans and animals, is considered the most infectious, causing pandemics. There is an emerging need for the accurate classification of the different influenza A virus (IAV) subtypes, elucidating their mode of infection, as well as their fast and accurate diagnosis. Notably, in recent years, oligomeric sequences (words) that are present in the pathogen genomes and entirely absent from the host human genome were suggested to provide robust biomarkers for virus classification and rapid detection. To this end, we performed updated phylogenetic analyses of the IAV hemagglutinin genes, focusing on the sub H1N1 and H5N1. More importantly, we applied in silico methods to identify minimum length “words” that exist consistently in the IAV genomes and are entirely absent from the human genome; these sequences identified in our current analysis may represent minimal signatures that can be utilized to distinguish IAV from other influenza viruses, as well as to perform rapid diagnostic tests.

## 1. Introduction

Influenza is a contagious disease that affects the respiratory tract and is caused by RNA viruses of the Orthomyxoviridae family. The species *Alphainfluenzavirus* correspond to Influenza A virus (IAV) (affecting animals and humans) and reunite all strains, whatever their origin and their antigenic properties. The three other species of influenza virus are *Betainfluenzavirus* (Influenza B virus; affecting humans), *Gammainfluenzavirus* (Influenza C virus; rarely affecting humans and pigs), and *Deltainfluenzavirus* (Influenza D virus; affecting cattle) [1,2].

The momentous consequences of this infection have arisen from the natural pool of influenza A (IAV) strains. This repository of viruses is actually an assortment of aquatic wild bird viruses—annotated as avian influenza viruses (AIV). They are characterized by varying pathogenicity, depending on the severity of the disease in the avian species they typically contaminate. Avian epizootics are frequent in farmed and wild birds, whilst millions of birds have died due to culling measures undertaken to control the infection or directly due to it. Strain A/H5 is the most frequent contaminator, but A/H7 is also both contagious and pathogenic. Transmission to humans is not a sine qua non but quite frequent, whilst many epidemics and pandemics have been reported even by Hippocrates in his books entitled *Epidemics* written in the 5th century BC [3].

The influenza contagion can be transmitted to everyone, although children and elders may be more vulnerable to certain strains, i.e., Vijaykrishna et al., investigating the history of the two strains of the influenza type B virus—called Victoria and Yamagata reported that between these disease outbreaks, Victoria viruses were more infective than the Yamagata viruses. Moreover, “Victoria viruses tended to infect younger patients than Yamagata viruses, which is thought to be due to differences in the molecules that help the viruses enter the cells of the respiratory tract” [4]. The route (mode) of transmission is via respiratory droplets (as soon as an infected person coughs or sneezes, or through saliva or mucus on unwashed hands) [5]. The symptoms of the disease are fever, cough, sore throat, muscle aches, headache, and/or fatigue, often leading to severe illness or even death (rarely in vaccinated populations).

The seasonal variation of influenza epidemics promotes preventive measures such as massive vaccinations, a measure that is well established worldwide. Yet, the latter, as well as other intervention strategies, need to be updated regularly, following the virus evolution and changes to the patterns of transmission. To this end, in molecular epidemiology, pathogens and host genomic analyses contribute to the:

- estimation of the disease dissemination within groups or communities as well as within and between countries,
- appraisal of the isolation options,
- contribution to future epidemics preparedness,
- tracing the pathogen(s) evolution.

The last is of utmost importance in the case of epidemics despite its phylogenetic analysis challenges (likelihood, biases, accuracy, topology, etc.). It has been established that the evolution of the influenza virus is gradual, and the adaptation of the vaccines to it has become a constant public health query. Recently, an ongoing research molecular target has been minimal absent words (MAWs). MAWs are oligomers of minimal length that are absent from the genome or proteome of an organism [6,7,8,9]. Although little light has been shed on the role of MAWs, they are assumed to have resulted from negative selection and have been of practical use in pesticide and/or drug target development [10,11], in forensics [12], or even in environmental monitoring and surveillance [6].

Research on viral diseases MAWs is limited and quite minimal in influenza, a known major public health problem. Thus, this study aims to fill in the gap in the literature and contribute by identifying possible MAWs within influenza types and (sub)types (intraspecies) and, hopefully, contribute to a better understanding of the disease history and spread across species (interspecies). In the present study, we refer to MAWs consistently conserved across viral genomes or particular groups of sequences (e.g., subtypes) as “prevalent relative MAW (PrMAW)”.

## 2. Materials and Methods

### 2.1. IAV Nucleotide Sequences

The complete nucleotide sequences of influenza A virus (IAV) were downloaded from the National Center for Biotechnology Information Virus (NCBI Virus) (https://www.ncbi.nlm.nih.gov/labs/virus/vssi/#/; accessed on 6 January 2025) collected from 1918–2024 for the human, pig and avian species. A total of 1229, 1185, 1167, 1825, 1173, 1460, 1293, and 1224 full-length IAV sequences were available for the polymerase PB2 (PB2), polymerase PB1 (PB1), polymerase PA (PA) and PA-X, hemagglutinin (HA), nucleocapsid protein (NP), neuraminidase (NA), matrix protein 2 (M2) and matrix protein 1 (M1), nuclear export protein (NEP) and nonstructural protein 1 (NS1) segments, respectively.

### 2.2. Phylogenetic İnference

The phylogenetic analyses were restricted to hemagglutinin (HA). Representative sequences from different geographical regions (i.e., continents and countries) and time periods (i.e., year) were selected for the IAV H1N1 and H5N1 HA segments; the sequences with >98% identity over their entire length were removed using the *dedupe.sh* script in the BBMap package (https://sourceforge.net/projects/bbmap/: accessed on 8 January 2025). The viral sequences were aligned with MAFFT v.7 using the iterative refinement method (FFT-NS-i option) [13]. Phylogenetic trees were constructed from the aligned sequences by employing the neighbor-joining method, which is based on a (agglomerative) hierarchical clustering algorithm implemented in the software package MEGA12 [14]. ModelTest [15] was used to estimate the best-fit model of nucleotide substitution. The robustness of the inferred phylogenetic trees was assessed by bootstrapping (1000 bootstrap pseudo-replicates).

### 2.3. Minimal Absent Words

The redundant (100% identity) human, swine, and avian IAV sequences were removed from the dataset. The non-redundant unaligned sequences (Table S1) were provided as input to EAGLE v2.2 (https://bioinformatics.ua.pt/software/eagle/; accessed on 16 January 2025) software tool, a comparative analysis software that identifies relative minimal non-existent words in target sequences against a reference sequence [16]. It effectively detects and analyzes unique motifs contained within a specified range size of fragments (k-mers), ranging from 11 bp to 16 bp, across large-scale genomic data [16,17]). The program constructs a model by dividing the target sequences into k-mers. Each k-mer from the target sequence is scanned with an alignment-free algorithm against the reference genome for its presence [17]. In our study, relative absent words were computed in the IAV genomes using the full GRCh38/hg38 human genome (https://genome.ucsc.edu/cgi-bin/hgGateway; accessed on 16 January 2025) downloaded from NCBI as reference. The graphical outputs generated by EAGLE are in shell script file format and visualized using the Gnuplot program. In addition, nucleotide and encoded protein sequences corresponding to the M1/M2 and PA genes/proteins were aligned with the European Bioinformatics Institute (EBI) GeneWise [18] (Text S1).

## 3. Results

### 3.1. Phylogenetic Analysis of IAV Sequences

#### 3.1.1. Phylogenetic Analysis of A/H1N1 Sequences

**Figure 1:**
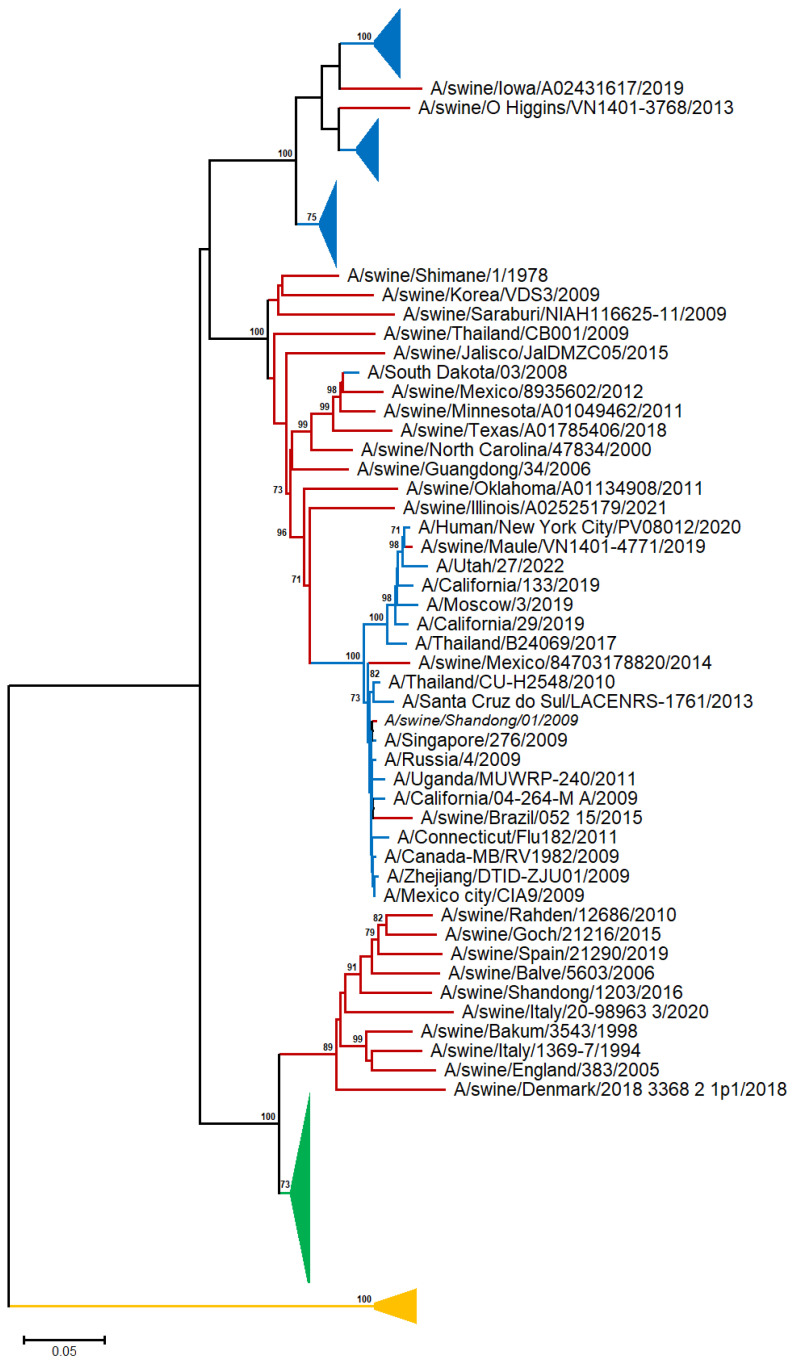
NJ-based tree (GTR + G model) of the H1N1 HA nucleotide sequences. Branches corresponding to human, swine, and avian sequences are shown in blue, red, and green color, respectively. The branch lengths indicate evolutionary distance. The tree is rooted using the IBV HA sequences as an outgroup (yellow color). Bootstrap values (>50%) are shown at the nodes. The scale bar at the lower left denotes the substitutions per nucleotide position. The major clades are collapsed for clarity.

A neighbor-joining (NJ) tree was reconstructed from 48 human, 28 swine, and 28 avian A/H1N representative sequences [19] (Table S1 and Figure 1 and Figure S1).

In the A/H1N1 phylogram, the avian sequences are clustered into a discrete, highly supported monophyletic clade, suggesting that there is no apparent spillover from birds to other species (swine and human). Although the human and swine A/H1N1 sequences exhibited a general tendency to cluster with those of the same organism, several swine sequences appeared to sort into the same clade with the human ones, in agreement with viral transmission to humans through direct contact with infected pigs [20].

The sequences collected before 1954 form a distinct, highly supported clade, consistent with the fact that the virus vanished for almost 20 years and reappeared in 1977, and the viral H1N1 serotype has undergone rapid antigenic evolution (‘antigenic drift’) [21]. The human sequences after 1977 are randomly distributed across the tree, suggesting common patterns of evolutionary change for the H1N1 serotype.

#### 3.1.2. Phylogenetic Analysis of A/H5N1 Sequences

**Figure 2:**
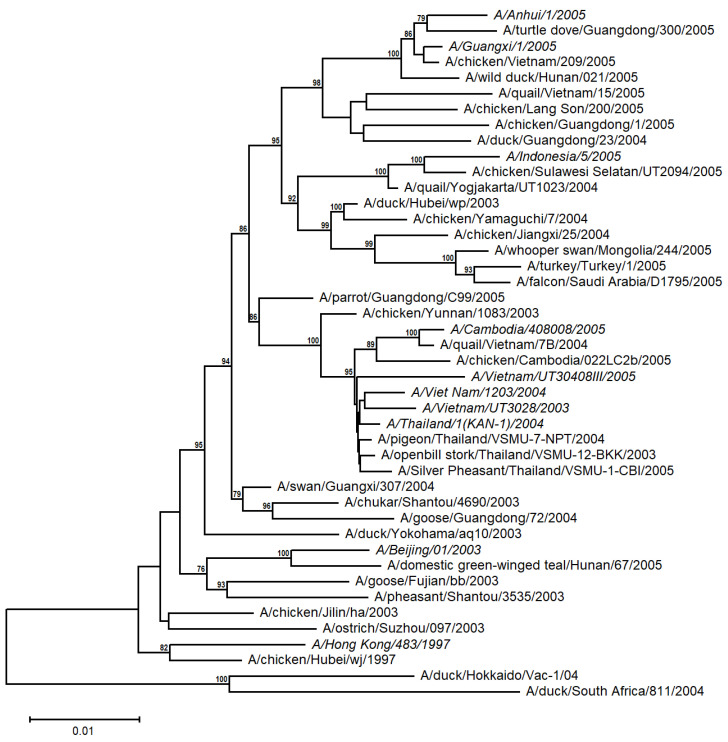
Unrooted NJ-based tree (HKY + G model) of the H5N1 HA nucleotide sequences. The human sequences are italicized. The branch lengths represent evolutionary distance. Bootstrap values (>50%) are shown at the nodes. The scale bar at the lower left denotes the substitutions per nucleotide position.

A distance-based tree was built from 10 human and 34 avian A/H5N1 sequences from the time periods 1997 and 2003 to 2005 (Table S1 and Figure 2). The human H5N1 sequences appear to be more closely related to the avian sequences from the corresponding geographical region (e.g., Indochina), with relatively high support values. This suggests that the A/H5N1 viruses evolved independently and differentiated into distinct populations in diverse avian families from different regions, irrespective of their emergence in the timetable, and then were transmitted to humans.

### 3.2. Prevalent Minimal Sequences

**Figure 3:**
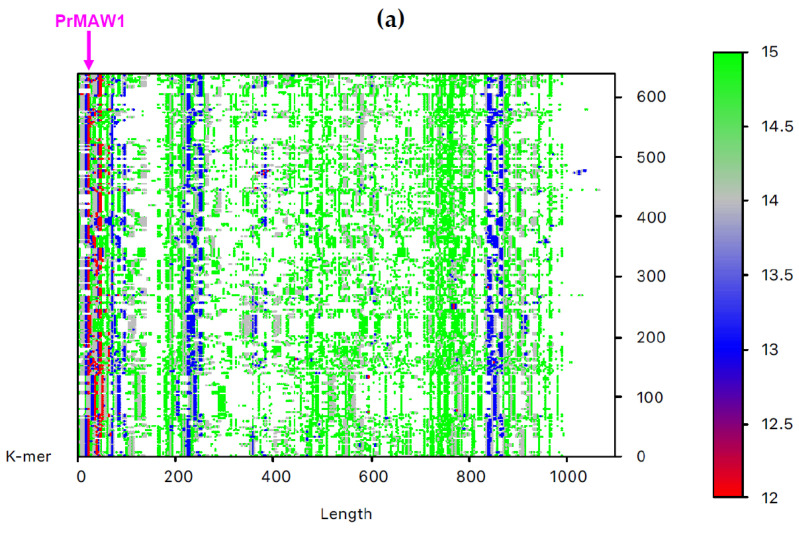

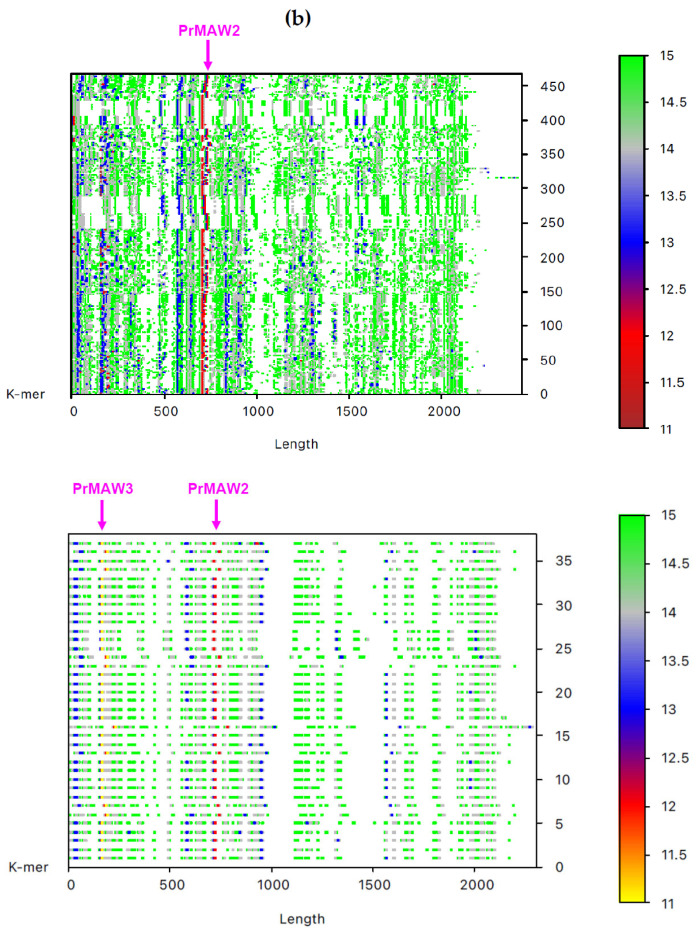
IAV PrMAWs relative to the entire human genome (indicated by arrows). Identification of PrMAWs in the unaligned IAV (**a**) M1/M2 and (**b**) PA nucleotide sequences. The vertical color scale bar represents minimal sequences of different lengths.

By employing the EAGLE software, we identified one 11-mer (yellow), 119 12-mers (red), and 2349 13-mers (blue) in 7733 non-redundant IAV sequences, which are not present in the influenza B virus (IBV) and C virus (ICV) genomes. From those, only three sequence motifs are prevalent and appear consistently at the same position, that is, the 12-letter motifs PrMAW1 (TCGAAACGTACG) and PrMAW2 (CGAACCGAACGG), as well as the 11-letter motif PrMAW3 (CGACGAACGAG) (Figure 3, arrows). PrMAW1 appears repeatedly across 466 nucleotide sequences of the M1/M2 gene; PrMAW2 and PrMAW3 are consistently present in 637 and 37 IAV sequences, respectively, that correspond to polymerase PA. Diverse IAV subtypes (H1N1, H1N2, H1N8, H2N2, H2N3, H2N7, H3N1, H3N2, H3N3, H3N6, H3N8, H4N2, H4N6, H4N8, H5N1, H5N2, H5N5, H5N6, H5N8, H5N9, H6N1, H6N2, H6N5, H6N6, H6N8, H7N1, H7N2, H7N3, H7N4, H7N7, H7N8, H7N9, H9N2, H9N7, H9N9, H10N2, H10N7, H10N8, H10N9, H11N9, H12N5, H12N8, H13N2, H13N6, H13N8) are represented in the two 12-mers. However, the 11-mer is persistent in the H7N9, H9N2, and H9N9 subtypes (Table S1 and Table 1). PrMAW1 is found in the N-terminal domain of the M1/M2 protein. PrMAW2 is included in the mid-region of the PA protein, whereas PrMAW3 is found in the N-terminus of PA (Text S1 and Table 1). The H9N9 serotype represents novel reassortant viruses with genes derived from both H9N2 and H7N9 [21]. This may be related to a common mode of co-infection with increased zoonotic potential.

## 4. Discussion

The influenza A and B virus genomes have eight negative-sense, single-stranded RNA segments, encoding 10 and 11 functional proteins, respectively, whereas influenza C and D comprise seven segments [1]. Influenza A viruses (IAV) are further divided into subtypes based on two surface glycoproteins with opposite functions, hemagglutinin (H) and neuraminidase (N) [22]. Wild aquatic birds are considered the primary reservoirs of IAV, but it also circulates in humans and pigs; the H1N1 and H3N2 IAV subtypes are currently circulating in humans [23]. Influenza B virus (IBV) and influenza C virus (ICV) are found mainly in humans; ICV is generally associated with mild respiratory disease [1,2,24]. Influenza D virus (IDV) can infect and/or be transmitted to various mammals, including cattle and pigs, without known zoonotic potential [1,2]. Influenza A and B viruses are widespread in humans and cause seasonal epidemics which vary in severity. Seasonal influenza (or “flu”) is caused by both IAV and IBV, which are mainly transmitted through small airborne droplets by coughing and sneezing or contaminated surfaces [5]. Flu is an acute respiratory infection that can spread efficiently, infect, and kill thousands of people each year around the world [5,22]. The IAV subtype H3N2 is manifested clinically with classic flu symptoms [6], and also a vaccine-resistant IAV H3N2 3C.2a1b.2a.2 subclade has been identified in wastewater from elementary schools in Las Vegas, USA [25]. Moreover, three subtypes of IAV have been identified in pigs (H1N1, H1N2, and H3N2), different from the human flu viruses, adapted to the swine physiology. The 2009 swine flu pandemic or A(H1N1)pdm09 is the third known flu pandemic caused by an IAV H1N1 virus, with 18,631 confirmed deaths [26,27]. The other two pandemics, namely the Spanish flu of 1918 and the 1977 Russian flu, killed about 50 million and 700,000 people [28].

Contrary to common assumptions, influenza epidemics have been described in the literature, ranging from the 5th century BC in Perinthos (an ancient Greek port on the Sea of Marmara, the inland sea that connects the Black to the Aegean Sea) and the Heian era in Japan (794–1185 CE), to the 20th-century’s repetitive outbreaks [3,18,29].

Epidemics of avian viruses have been numerous, with the most recent [28]

- In 1357, when the influenza term first appeared.
- In 1510, a pandemic originating from Asia, possibly China, spread through Africa to Europe; coccolucio (in Italian) or coqueluche (in French).
- In 1889–1890, the Asiatic flu.
- In 1918, in Spain, H1N1 infected 30% of the world population, killing millions of people.
- In 1933, the virus was discovered.
- In 1957, when H2N2 caused the so-called “Asian flu” in Asia.
- In 1967, H3N2 brought about the Hong Kong flu.
- In 1968, the Hong Kong flu invaded Japan.
- In 1977–1979, H1N1 gave rise to the Russian flu pandemic (in children).
- In 1996, when H5N1 was isolated in a farmed goose in China.
- In 1997, an H5 substrain in poultry was identified in Hong Kong.
- In 2003, H5N1 was spread in poultry across Southeastern Asia.
- In 2004, H5N1 expanded further in Eastern Asia, infecting poultries, other birds, and even felidae.
- In 2005, H5N1 was found in Turkey, Romania, and Ukraine (Middle East and Europe).
- In 2009, the swine flu passed on to Japan after Mexico and North America.
- In 2013, H7N9 had been isolated in China, disseminating among humans effectively. As vaccines against this strain were produced and applied, the population reached herd immunity; thus, the epidemic is considered eradicated since 2019.
- In 2014, H5N8 led to outbreaks in Europe in wild birds, mostly in poultry.
- In 2015–2016, H1N1 predominated in Europe.
- In 2016–2018, H3N2 dominated in Europe.
- In 2018–2019, H1N1pdm09, A/H3N2 prevailed in Western countries.
- In 2019–2020, A/H1N1pdm09, B were mainly isolated across Western countries.
- In 2021–2022, A/H3N2 was spread along with SARS-CoV-2.
- In 2022–2024, A/H1N1pdm09 predominated, although isolation (due to Coronavirus co-contagion) and wide vaccination eventually decreased the incidence of the disease.
- Typical seasonal flu.

The above history dictates that genomic epidemiology analyses are imperative for surveillance and preparedness reasons.

Phylogenetics allow us to trace the evolutionary trajectory of diverse influenza virus variants in a timetable; phylogeography enables the study of the geographical distribution of viral lineages [30]. It underlines the fact that humans became more vulnerable to zoonotic infections after their frequent interactions with domesticated birds and pigs [31].

Estimation of phylogenetic relationships among genes and species traditionally involves multiple sequence alignment. As sequencing data volume escalates, the urgent need for faster yet accurate alignment-free methods arises equally. Anjum et al. (2023) recently proved that CD-MAWS resolved phylogenetic relationships similar to or better than state-of-the-art alignment-free methods such as Mash, Skmer, Co-phylog, and kSNP3 on diverse biological datasets such as mammalian mtDNA, bacterial and viral genomes [32].

Herein, neighbor-joining, a straightforward method of phylogenetic inference, where the branch lengths of the tree correspond as closely as possible to the observed set of distances between pairs of sequences [19], was applied. Since viruses accumulate mutations (resulting from biochemical processes) and not substitutions (a slow and gradual evolutionary process), which enables them to evolve quite rapidly, we considered NJ an optimal method to detect any changes in the composition of the viral nucleotide or amino acid sequences.

The recent extension of the molecular analyses to MAWs (broad term including nullomers and longer sequences that become present after deleting either their leftmost or rightmost letter) [8], nullomers (“the shortest possible absent motifs in a species”) and primes (“the shortest sequences that are not found across all known species”) [9] appears promising if conducted prudently by minimizing analyses biases, increasing accuracy and plausibility in the disease mutation history or evolution.

It is established that there are higher numbers of larger-sized k-mers (i.e., DNA 15-mers are found more frequently than 11-mers) [7,8,9]. In a previous study, Silva et al. (2015) [16] detected three 12-letter minimal relative absent words that are consistently present in two protein-coding genes of the Ebola virus and completely absent from the human host genome. Moreover, Pratas and Silva (2020) identified four persistent MAWs in the SARS-CoV-2 genome [17]. We hereby investigated all influenza serotypes (A, B, C, D) identifying (i) PrMAWs that are present only in the IAV sequences, but (ii) absent from the human genome.

More explicitly, we identified three sequences that are entirely absent from the human genome but prevalent in the IAV genomes, one with size 11 and two with size 12. One 12-mer PrMAW1 was found in the sequences that correspond to the M1/M2 protein; M2 is a proton-gated proton channel that is essential for IAV propagation; M2 also interacts with M1, the most abundant virus protein that mediates virion assembly by forming an endoskeleton underneath the virus membrane [15,33]. Two motifs, PrMAW2 and PrMAW3, are present across the sequences that encode the non-structural polymerase PA, a subunit of the heterotrimeric RNA-dependent RNA polymerase implicated in the transcription and replication of the viral RNA [34]. Moreover, PrMAW3 might be positively selected in H7N9, H9N2, and H9N9, presumably in order to drive specific segment combinations during genomic reassortment in IAV [35].

The sequence motifs most missing from the human genome were found to be of 11 bp length [6,9]. It is suggested that the rare occurrence and absence of certain motifs (in humans and mammals) originated from mutations and not from selective pressures [9,36]. Indeed, Acquisti and colleagues had established that “the hypermutability of CpG dinucleotides, rather than the natural selection against the nullomer sequences, is likely the reason for the phenomenal event of short sequence motifs becoming nullomers. Furthermore, many reported human nullomers differ by only one nucleotide, which reinforces the role of mutation in the evolution of the constellation of nullomers in populations and species” [7]. Moreover, viral sequences tend to mimic those of their respective hosts (“viral mimicry”) in order to evade the host’s immune responses [37].

Therefore, the PrMAWs present in IAV sequences could be considered as potential surveillance as well as therapeutic targets, as Hampikian and Andersen demonstrated that the body’s “defense” system naturally recognizes nullomers and prepares for oligomers that may arise from them, further explaining variation in human vulnerability [9].

Finally, we should note the limitations of this study: it is restricted to the hemagglutinin antigen and did not examine the neuraminidase antigen. Since 1969, the World Health Organization has recommended virtual preparedness for future flu pandemics originating from one of the 16 HA antigen (subtypes) mutations that have been found in avian and/or mammalian species [28,38]. Thus, the hereby identified PrMAW surveillance is restricted to human hosts, and its application for other influenza viruses in other hosts needs to be investigated.

## 5. Conclusions

The results of this study corroborate and highlight that (a) The H9N9 subtype has apparently emerged from the genetic reassortment between H9N2 and H7N9; (b) human vulnerability might be attributed to the herein-identified three sequence motifs that persist at the same position (i.e. the 12-letter motifs PrMAW1 (TCGAAACGTACG) and PrMAW2 (CGAACCGAACGG), as well as the 11-letter motif PrMAW3 (CGACGAACGAG)) found in various IAV strains but abstain from human motifs. Thus, they could be taken into consideration for PCR-based rapid diagnosis or even targeted therapy designs.

## Figures and Tables

**Table 1:** IAV PrMAWs and encoded peptides.

Segment	Sequences Analyzed	Sequences Detected	Subtypes/Subtypes	PrMAW	Motif	Peptide
M1/M2	711	637	H1-H7, H9-H13	PrMAW1	TCGAAACGTACG	xETYx
PA and PA-X	588	466	H1-H7, H9-H13	PrMAW2	CGAACCGAACGG	xEPNx
PA and PA-X	38	37	H7N9, H9N2, H9N9	PrMAW3	CGACGAACGAG	xDERx

## Data Availability

No new data were created.

## Supplementary Materials

The following supporting information can be downloaded at: https://www.mdpi.com/article/10.3390/v17050659/s1, Table S1: IAV sequence analyzed in the current study; Text S1: Nucleotide and protein alignments of the *M1/M2* and *PA* genes. Regions corresponding to the identified PrMAWs are indicated; Figure S1: NJ-based tree (GTR + G model) of the H1N1 HA nucleotide sequences. The conventions are the same as in Figure 1.

## Abbreviations

The following abbreviations are used in this manuscript:

HA	hemagglutinin
IAV	Influenza A virus
IBV	Influenza B virus
ICV	Influenza C virus
M1	matrix protein 1
M2	matrix protein 2
NA	neuraminidase
NEP	nuclear export protein
NP	nucleocapsid protein
NS1	nonstructural protein 1
PA	nonstructural protein 1
PrMAW	prevalent minimal absent words
