# Supplementary material for: Novel Minimal Absent Words Detected in Influenza A Virus
Source: Viruses. 2025 Apr 30;17(5):659. doi: 10.3390/v17050659 (PMC12116108; doi:10.3390/v17050659)
Supplement: Supplementary file 1 [file viruses-17-00659-s001.zip › TextS1.pdf]

Segment 7; matrix protein 2 (M2) and matrix protein 1 (M1):

```
YP_009118623.1     1 MSLLTEVETYVLSIIPSGPLKAEIAQRLESVFAGKNTDLEALMEWLKTR
                     MSLLTEVETYVLSIIPSGPLKAEIAQRLESVFAGKNTDLEALMEWLKTR
                     MSLLTEVETYVLSIIPSGPLKAEIAQRLESVFAGKNTDLEALMEWLKTR
NC_026431.1        1 aaccagggatgctaactgccaggagcacgagtggaaagcggcagtcaaa
                     tgttcatacattcttccgctacatcagtagttcgaacatacttagtacg
                     gttacgcagctttccgacccacgcggagatctaagcattgtcgagagaa
                         PrMAW1

YP_009118623.1    50 PILSPLTKGILGFVFTLTVPSERGLQRRRFVQNALNGNGDPNNMDRAVK
                     PILSPLTKGILGFVFTLTVPSERGLQRRRFVQNALNGNGDPNNMDRAVK
                     PILSPLTKGILGFVFTLTVPSERGLQRRRFVQNALNGNGDPNNMDRAVK
NC_026431.1      148 cattccaagatgtgtacagcagcgcccactgcagcagaggcaaagagga
                     cttcctcagttgtttctctcgaggtagggttaactagagacaatagcta
                     acgatgtgataatgcgccgctgaaggtactcatcatgtgcgccgtaata

YP_009118623.1    99 LYKKLKREITFHGAKEVSLSYSTGALASCMGLIYNRMGTVTTEAAFGLV
                     LYKKLKREITFHGAKEVSLSYSTGALASCMGLIYNRMGTVTTEAAFGLV
                     LYKKLKREITFHGAKEVSLSYSTGALASCMGLIYNRMGTVTTEAAFGLV
NC_026431.1      295 ctaacaagaatcggaggtcattaggcgatagcataaagagaagggtgcg
                     taaatagatctagcaatctgaccgctcggtgttaagtgctccacctgtt
                     acggcaaaagctgcgggaactattatctcgccaccggaagcaattttag

YP_009118623.1   148 CATCEQIADSQHRSHRQMATTTNPLIRHENRMVLASTTAKAMEQMAGSS
                     CATCEQIADSQHRSHRQMATTTNPLIRHENRMVLASTTAKAMEQMAGSS
                     CATCEQIADSQHRSHRQMATTTNPLIRHENRMVLASTTAKAMEQMAGSS
NC_026431.1      442 tgatgcaggtccctcacagaaaaccaacgaaagcgaaagagagcaggta
                     gccgaatcacaagcagatccccacttgaaagtttcgcccactaatcgcg
                     tcttagtttagtgtcaggttcctaacgtacagggtctgagtgaggtagt

YP_009118623.1   197 EQAAEAMEVANQTRQMVHAMRTIGTHPSSSAGLKDDLLENLQAYQKRMG
                     EQAAEAMEVANQTRQMVHAMRTIGTHPSSSAGLKDDLLENLQAYQKRMG
                     EQAAEAMEVANQTRQMVHAMRTIGTHPSSSAGLKDDLLENLQAYQKRMG
NC_026431.1      589 gcggggagggacaacagcgaaaagaccataggcaggccgatcgtcacag
                     aaccactatcaacgattactgctgcacgcgcgtaaattaatacaaagtg
                     agaggcggtttgtgggatagattgtttcctttgatcttatggccggaga

YP_009118623.1   246 VQMQRFK
                     VQMQRFK
                     VQMQRFK
NC_026431.1      736 gcaccta
                     tatagta
                     ggggacg
```

Segment 3; polymerase PA (PA) and PA-X protein (PA-X):

```
YP_009118473.1      1 MEDFVRQCFNPMIVELAEKAMKEYGEDPKIETNKFASICTHLEVCFMYS
                      MEDFVRQCFNPMIVELAEKAMKEYGEDPKIETNKFASICTHLEVCFMYS
                      MEDFVRQCFNPMIVELAEKAMKEYGEDPKIETNKFASICTHLEVCFMYS
NC_026424.1         1 aggtgccttacaaggcggagaagtgggcaagaaatgtatactggttatt
                      taattgagtactttatcaactaaagaacatacaatcctgcatatgttac
                      gactgagcctagccgtgagagaatgatgacaacacaaacacaacccgct

YP_009118473.1     50 DFHFIDERGESTIIESGDPNALLKHRFEIIEGRDRTMAWTVVNSICNTT
                      DFHFIDERGESTIIESGDPNALLKHRFEIIEGRDRTMAWTVVNSICNTT
                      DFHFIDERGESTIIESGDPNALLKHRFEIIEGRDRTMAWTVVNSICNTT
NC_026424.1       148 gtctaggcggtaaagtggcagccacctgaaggagcaagtaggaaataaa
                      atattaaggaccttacgacacttaagtattaggagctcgcttagtgacc
                      tcccccaacaataaatctatgggacataacagacaagcgaggttcccca
                           PrMAW3

YP_009118473.1     99 GAEKPKFLPDLYDYKENRFIEIGVTRREVHIYYLEKANKIKSEKTHIHI
                      GAEKPKFLPDLYDYKENRFIEIGVTRREVHIYYLEKANKIKSEKTHIHI
                      GAEKPKFLPDLYDYKENRFIEIGVTRREVHIYYLEKANKIKSEKTHIHI
NC_026424.1       295 gggacatccgctgtagactagaggaaaggcattcgagaaaatgaacaca
                      gcaacattcataaaaaagttatgtcggatataataacaatacaacatat
                      acaacatcgtgtccgactctataggggaccatcagactaaacggacctt

YP_009118473.1    148 FSFTGEEMATKADYTLDEESRARIKTRLFTIRQEMASRGLWDSFRQSER
                      FSFTGEEMATKADYTLDEESRARIKTRLFTIRQEMASRGLWDSFRQSER
                      FSFTGEEMATKADYTLDEESRARIKTRLFTIRQEMASRGLWDSFRQSER
NC_026424.1       442 tttagggagaaggtacgggaagaaaaactaaacgagaagctgttcctga
                      tctcgaatccacaactaaaggcgtacgttctgaatcgggtgactgacag
                      tactaaggccaatcttcaacgaacacggccaggagccgtagtcttgtaa

YP_009118473.1    197 GEETIEERFEITGTMRRLADQSLPPNFSSLENFRAYVDGFEPNGCIEGK
                      GEETIEERFEITGTMRRLADQSLPPNFSSLENFRAYVDGFEPNGCIEGK
                      GEETIEERFEITGTMRRLADQSLPPNFSSLENFRAYVDGFEPNGCIEGK
NC_026424.1       589 gggaaggatgaagaacacggcacccattacgatagtgggtgcagtagga
                      gaactaagtatcgctggtcaagtccatcgtaatgcatagtacaggtaga
                      caaataaatacaacgcgtccatcagcccctactactgtacagccctgcg
                                                              PrMAW2

YP_009118473.1    246 LSQMSKEVNARIEPFLRTTPRPLRLPNGPPCSQRSKFLLMDALKLSIED
                      LSQMSKEVNARIEPFLRTTPRPLRLPNGPPCSQRSKFLLMDALKLSIED
                      LSQMSKEVNARIEPFLRTTPRPLRLPNGPPCSQRSKFLLMDALKLSIED
NC_026424.1       736 ctcataggagaagctcaaaccccatcagccttcctattcaggcataagg
                      tcatcaatacgtacttgcccgctgtcagccgcagcattttactatgtaa
                      ttggaaagccatgataaaaactcagttgtcctgggacgggttgaactgc

YP_009118473.1    295 PSHEGEGIPLYDAIKCMKTFFGWKEPNIIKPHEKGINPNYLLTWKQVLA
                      PSHEGEGIPLYDAIKCMKTFFGWKEPNIIKPHEKGINPNYLLTWKQVLA
                      PSHEGEGIPLYDAIKCMKTFFGWKEPNIIKPHEKGINPNYLLTWKQVLA
NC_026424.1       883 cacggggacctggaataaattgtagcaaaaccgagaacatccatacgcg
                      cgaagagtctaactagtacttggaacattacaaagtacaattcgaattc
                      gccagggagattgcacgagccggagcctcaatgacaccttcgtggggaa

YP_009118473.1    344 ELQDIENEEKIPRTKNMKKTSQLKWALGENMAPEKVDFEDCKDVNDLKQ
                      ELQDIENEEKIPRTKNMKKTSQLKWALGENMAPEKVDFEDCKDVNDLKQ
                      ELQDIENEEKIPRTKNMKKTSQLKWALGENMAPEKVDFEDCKDVNDLKQ
```

```
NC_026424.1     1030 gccgagaggaacaaaaaaaaactatgcggaagcgaggtggtaggagtac
                     ataataaaaatcgcaataacgatagctgaatccaatataagaataataa
                     atgctataggtagagcggaacaaggactgcgaggggctgtcatcccgag

YP_009118473.1   393 YNSDEPEPRSLACWIQNEFNKACELTDSSWVELDEIGEDVAPIEHIASM
                     YNSDEPEPRSLACWIQNEFNKACELTDSSWVELDEIGEDVAPIEHIASM
                     YNSDEPEPRSLACWIQNEFNKACELTDSSWVELDEIGEDVAPIEHIASM
NC_026424.1     1177 taaggcgcatcgttacagtaagtgcagtatggcggagggggcagcagaa
                     aagaacacgctcggtaaataacgatcacggtataatgaatcctaatcgt
                     ccttgagcaaaatgcgtaccggtagtcacgaattaagattcacactacg

YP_009118473.1   442 RRNYFTAEVSHCRATEYIMKGVYINTALLNASCAAMDDFQLIPMISKCR
                     RRNYFTAEVSHCRATEYIMKGVYINTALLNASCAAMDDFQLIPMISKCR
                     RRNYFTAEVSHCRATEYIMKGVYINTALLNASCAAMDDFQLIPMISKCR
NC_026424.1     1324 acattagggtctagagtaaaggtaaagtcagttggaggtccacaaaata
                     ggaatccatcaggccaattagtataccttaccgcctaatattcttgagg
                     agcttaaggcccgttataggagcatatgctattacgtctagtagatata

YP_009118473.1   491 TKEGRRKTNLYGFIIKGRSHLRNDTDVVNFVSMEFSLTDPRLEPHKWEK
                     TKEGRRKTNLYGFIIKGRSHLRNDTDVVNFVSMEFSLTDPRLEPHKWEK
                     TKEGRRKTNLYGFIIKGRSHLRNDTDVVNFVSMEFSLTDPRLEPHKWEK
NC_026424.1     1471 aaggacaaactgtaaagatctaagagggatgaagttcagcatgccatga
                     caagggacatagtttaggcatgaacattattgtatctcacgtacaagaa
                     caaaagaacgtactaaaattggttccgactatgatctccaggaatagag

YP_009118473.1   540 YCVLEIGDMLLRTAVGQVSRPMFLYVRTNGTSKIKMKWGMEMRRCLLQS
                     YCVLEIGDMLLRTAVGQVSRPMFLYVRTNGTSKIKMKWGMEMRRCLLQS
                     YCVLEIGDMLLRTAVGQVSRPMFLYVRTNGTSKIKMKWGMEMRRCLLQS
NC_026424.1     1618 ttgcgaggacccagggcgtacatctgaaagataaaaatgagaactccct
                     agttatgatttgcctgatcgctttatgcagccatataggtatgggttac
                     ttttaagcgcgataacagaacgtgtgactgccgcggagtgagaccttat

YP_009118473.1   589 LQQIESMIEAESSVKEKDLTKEFFENKSETWPIGESPKGVEEGSIGKVC
                     LQQIESMIEAESSVKEKDLTKEFFENKSETWPIGESPKGVEEGSIGKVC
                     LQQIESMIEAESSVKEKDLTKEFFENKSETWPIGESPKGVEEGSIGKVC
NC_026424.1     1765 cccagaaagggttgagagcaagttgaatgatcaggtcagggggtagagt
                     taatagttacacctaaaatcaattaaacacgctgaccagtaagctgatg
                     cagtgcgtatacccagacgcaactacaaaagatagataaggatccgggc

YP_009118473.1   638 RTLLAKSVFNSLYASPQLEGFSAESRKLLLIVQALRDNLEPGTFDLEGL
                     RTLLAKSVFNSLYASPQLEGFSAESRKLLLIVQALRDNLEPGTFDLEGL
                     RTLLAKSVFNSLYASPQLEGFSAESRKLLLIVQALRDNLEPGTFDLEGL
NC_026424.1     1912 aatcgatgtaactgtcccggttggtaacccagcgcagacgcgatgcggc
                     gcttcacttagtacccatagtccacgatttttactgaatacgctatagt
                     acaaaatacccatatgacggcatagaagacttggtgtcgatactttaga

YP_009118473.1   687 YEAIEECLINDPWVLLNASWFNSFLTHALR
                     YEAIEECLINDPWVLLNASWFNSFLTHALR
                     YEAIEECLINDPWVLLNASWFNSFLTHALR
NC_026424.1     2059 tggaggtcaagctgtcagtttattcacgca
                     aactaagttaacgtttaccgtacttcactg
                     taacggcgtttcgtgttatgcccccataaa
```
