# Supplementary figures and images for: Novel Minimal Absent Words Detected in Influenza A Virus
Source: Viruses. 2025 Apr 30;17(5):659. doi: 10.3390/v17050659 (PMC12116108; doi:10.3390/v17050659)

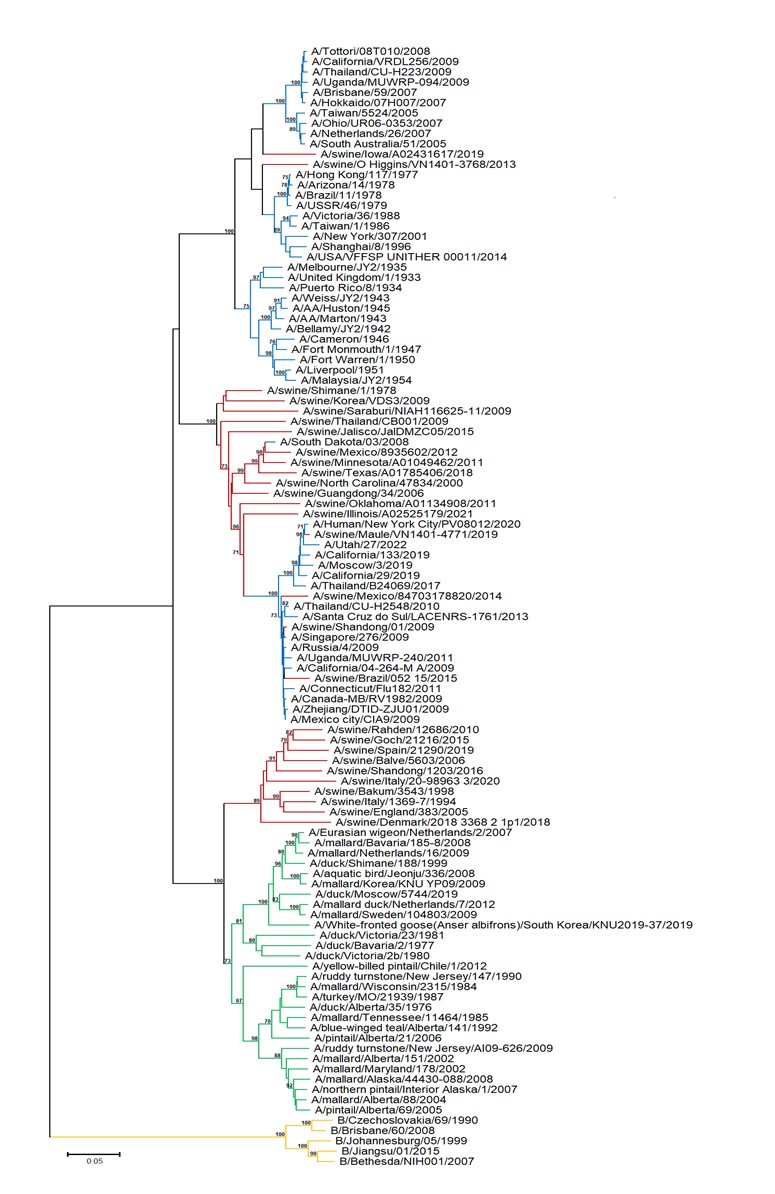

Supplement: Supplementary file 1 [file viruses-17-00659-s001.zip › Figure S1.jpg]
